# Novel Fabrication and Enhanced Photocatalytic MB Degradation of Hierarchical Porous Monoliths of MoO_3_ Nanoplates

**DOI:** 10.1038/s41598-017-02025-3

**Published:** 2017-05-12

**Authors:** Yang Liu, Peizhong Feng, Zhang Wang, Xinyang Jiao, Farid Akhtar

**Affiliations:** 1 School of Materials Science and Engineering, China University of Mining and Technology, Xuzhou, 221116 P. R. China; 2 Division of Materials Science, Luleå University of Technology, Luleå, 97187 Sweden

## Abstract

Porous monoliths of MoO_3_ nanoplates were synthesized from ammonium molybdate (AHM) by freeze-casting and subsequent thermal treatment from 300 to 600 °C. Pure orthorhombic MoO_3_ phase was obtained at thermal treatment temperature of 400 °C and above. MoO_3_ monoliths thermally treated at 400 °C displayed bimodal pore structure, including large pore channels replicating the ice crystals and small pores from MoO_3_ sheets stacking. Transmission electron microscopy (TEM) images revealed that the average thicknesses of MoO_3_ sheet were 50 and 300 nm in porous monoliths thermally treated at 400 °C. The photocatalytic performance of MoO_3_ was evaluated through degradation of methylene blue (MB) under visible light radiation and MoO_3_ synthesized at 400 °C exhibited strong adsorption performance and best photocatalytic activity for photodegradation of MB of 99.7% under visible illumination for 60 min. MoO_3_ photocatalyst displayed promising cyclic performance, and the decolorization efficiency of MB solution was 98.1% after four cycles.

## Introduction

Transition metal oxide semiconductors (TMOs)^1, 2^ are viable materials due their electronic band structures, physical properties and stability in demanding chemical environments, and therefore have been used in different applications such as gas sensors, resonators and high-efficiency catalysts^3–6^. TMOs, such as TiO_2_, WO_3_, MoO_3_ and CeO_2_, are photocatalysts^7–9^ and decompose organic pollutant, such as phenol^10^, methyl orange^11^, rhodamine B^12^ and methylene^13^ MoO_3_ is a n-type semiconductor and exists in three main crystal structures: orthorhombic (α-MoO_3_), monoclinic (β-MoO_3_) and hexagonal (h-MoO_3_)^14–16^. Particularly, α-MoO_3_ has been considered as a potential photocatalyst material in terms of its anisotropic layered structure^17^, where highly asymmetrical [MoO_6_] octahedrons assemble into a bilayer in such a manner that certain octahedrons share four corners to form a plane, further combining with another plane by sharing octahedral edges along the [001] direction and all the bilayers stack up along the [010] direction with weak van der Waals forces^18–20^. Compared with the bulk counterpart, significantly large surface area and high aspect ratio could be expected in the 1D nanostructure^21^.

Numerous methods have been developed to synthesize MoO_3_, such as magnetron sputtering^22^, chemical precipitation^23^, hydrothermal synthesis^24^, electrocatalytic oxidation^25, 26^ and physical vapor deposition^27^. Sara Alizadeh *et al*.^28^ synthesized MoO_3_ through a facile salt method using NH_4_NO_3_ as a molten salt. X. S. Yuan *et al*.^29^ synthesized MoO_3_ · 0.5H_2_O via a room-temperature aqueous chemical method. Zhang *et al*.^30^ synthesized 2D MoO_3_ nanosheets by liquid exfoliation method. Though various morphologies of MoO_3_ have been synthesized, the synthesis processes were complex and not in environmental protection. In recent years, freeze-drying has been explored as a unique route to produce novel porous materials. Freeze-drying using water offers advantages such as water is an environment-friendly solvent and the use of ice crystals as porogens is green and sustainable. Moreover, the growth speed and orientation of the ice crystals can be controlled to obtain unidirectional porous scaffolds. More importantly, by changing variables during freezing, it is possible to produce materials with a variety of pore morphologies and nanostructures^31^.

In this study, hierarchically porous monoliths of α-MoO_3_ nanoplates of high purity were synthesized through the combination of the freeze-drying and thermal treatment, and the phase composition and microstructure were investigated. The photocatalytic activity of the monoliths was evaluated through the degradation of MB under visible radiation. The results showed that the as-synthesized MoO_3_ exhibited high-efficiency catalytic as well as adsorption performance, and the decomposition efficiency of 30 mg/L MB was 98.8% under illumination for 30 min, which was far superior to the decomposition efficiencies reported in literature.

## Results and Discussion

### Phase analysis and morphology observation

**Figure 1:**
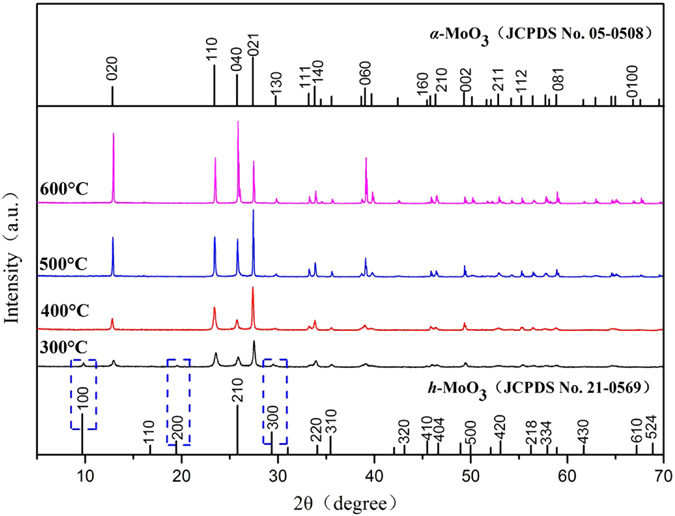
X-ray diffraction patterns of MoO_3_ synthesized at different thermal treatment temperatures.

X-ray diffraction patterns (XRD) of porous ammonium molybdate (AHM) after thermal treatment between 300 and 600 °C in Fig. 1 shows the formation of α-MoO_3_. Porous AHM, thermally treated at 300 °C shows the presence of h-MoO_3_ diffraction peaks at the 2θ of 9.69°, 19.45° and 29.355° in addition to diffraction peaks of α-MoO_3_. Porous AHM thermally treated at 400 °C shows that all the diffraction peaks of the synthesized products correspond to only α-MoO_3_. On increase in thermal treatment temperature of porous AHM to 500 and 600 °C, the intensities of (020), (040) and (060) diffraction peaks increased (Fig. 1). It suggests that the α-MoO_3_ crystals grow preferentially along (0k0) direction with increase of thermal treatment temperature. The average crystallite sizes of α-MoO_3_ obtained at different thermal treatment temperature are estimated by using Scherrer’s equation (L = 0.89λ/β cos θ)^32^ and are shown in Table 1.

**Table 1:** Average crystallite thicknesses of synthesized MoO_3_ crystals.

Thermal treatment temperature (°C)	300	400	500	600
Average crystallite thickness (nm)	27.8	45.4	>100	>100

**Figure 2:**
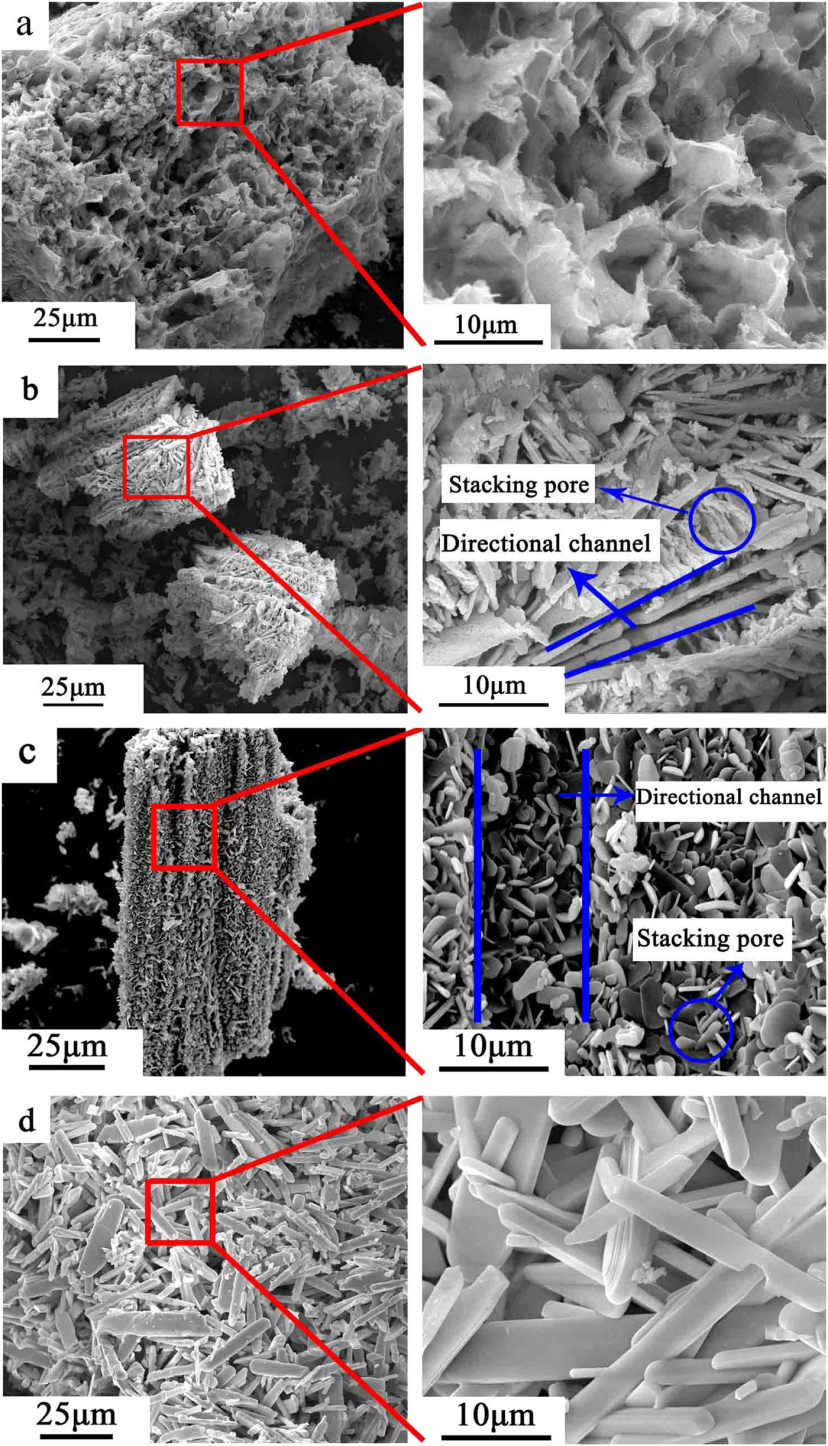
SEM images of MoO_3_ synthesized at different sintering temperature: (**a**) 300 °C, (**b**) 400 °C, (**c**) 500 °C, (**d**) 600 °C.

**Figure 3:**
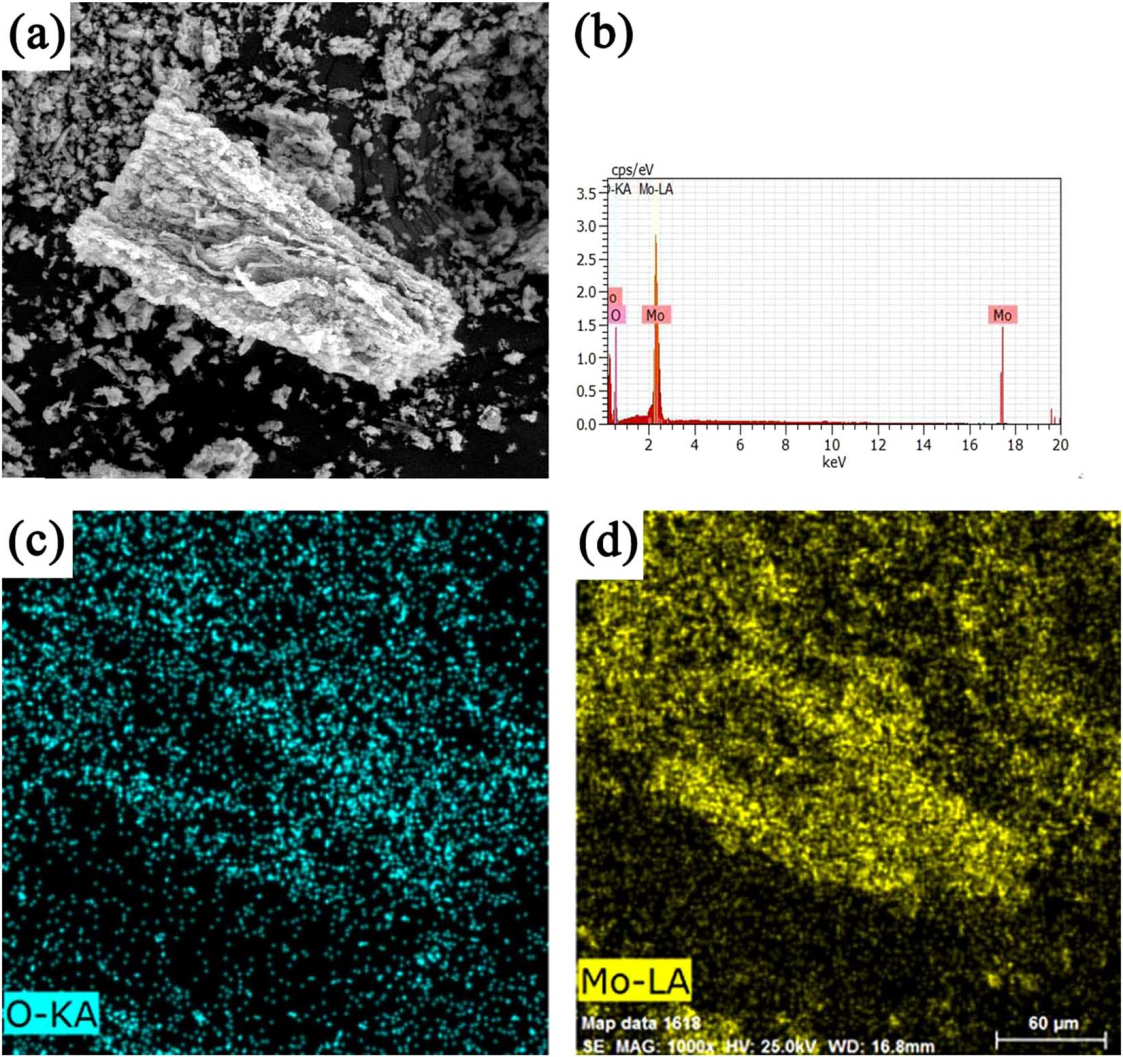
(**a**) SEM of the MoO_3_ synthesized at 400 °C, (**b**) EDS spectrum, (**c,d**) Elemental mapping of Mo and O.

Figure 2 shows the morphology of as-synthesized α-MoO_3_ treated at different temperatures. MoO_3_ synthesized at 300 °C is composed of foam-like cellular structure (Fig. 2a). At low thermal treatment temperature, the α-MoO_3_ crystals show lower crystallinity and do not show rod-like or sheet-like morphology typical of α-MoO_3_^33^. On increase of thermal treatment temperature to 400 °C, bimodal pore structure is visible (Fig. 2b) including large porous channels replicating from ice sublimation and small pores originating from stacking of α-MoO_3_ nanosheets. As shown in Fig. 2c, when the thermal treatment temperature is further raised to 500 °C, α-MoO_3_ sheets show typical crystalline morphology with the average sheet thickness of 300 nm. However, when the thermal treatment temperature increased to 600 °C, α-MoO_3_ morphology changed greatly (Fig. 2d). It can be clearly seen that α-MoO_3_ powder present a belt-like structure with an average thickness of 2 μm and a length of about 25 μm. This morphology evolved because of the growth of α-MoO_3_ crystal along both a axis and b axis. SEM results confirm that the synthesis temperature has a significant impact on the morphologies of α-MoO_3_. Figure 3 shows the energy dispersive spectrum (EDS) and elemental mapping of porous MoO_3_ synthesized at 400 °C and demonstrate the homogeneous distribution of Mo and O elements.

**Figure 4:**
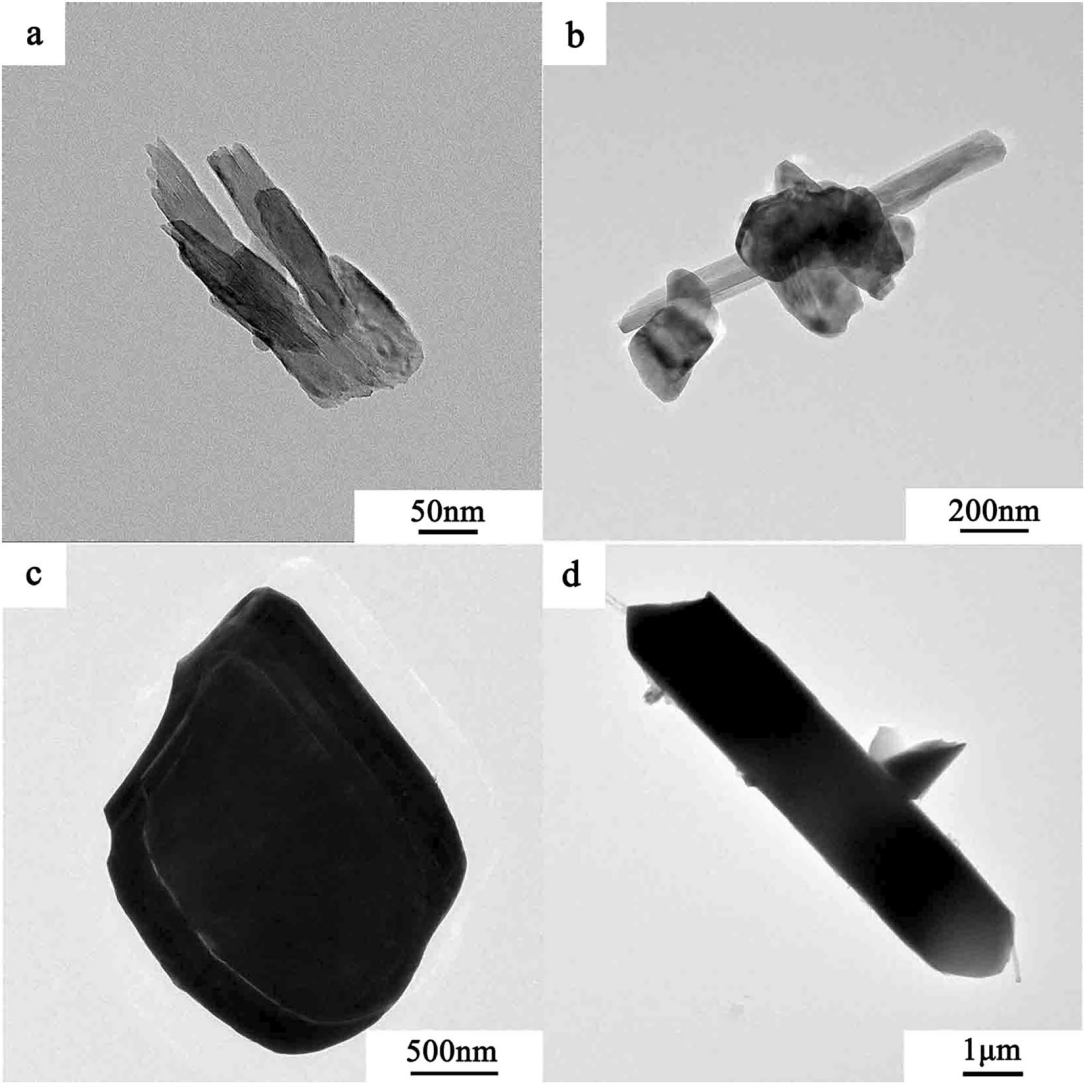
TEM images of α-MoO_3_ synthesized at (**a**) 300 °C, (**b**) 400 °C, (**c**) 500 °C and (**d**) 600 °C.

Transmission electron micrographs (TEM) of the MoO_3_ single crystals in Fig. 4 show that α-MoO_3_ nanoplates synthesized at 300 °C have irregular morphology with an average diameter of 200 nm and thickness between 20 and 40 nm. Similarly, α-MoO_3_ synthesized at 400 °C (Fig. 4b) exhibited plate thickness of about 50 nm. When the temperature rises to 500 °C, the crystals further grow to an average thickness of 300 nm (Fig. 4c). However, α-MoO_3_ nanoplates change to nanorods at the thermal treatment temperature of 600 °C (Fig. 4d). The nanorods are 7 μm long. TEM results further confirm that synthesis temperature has a significant impact on the crystal size and morphology of α-MoO_3_.

### FT-IR analysis

**Figure 5:**
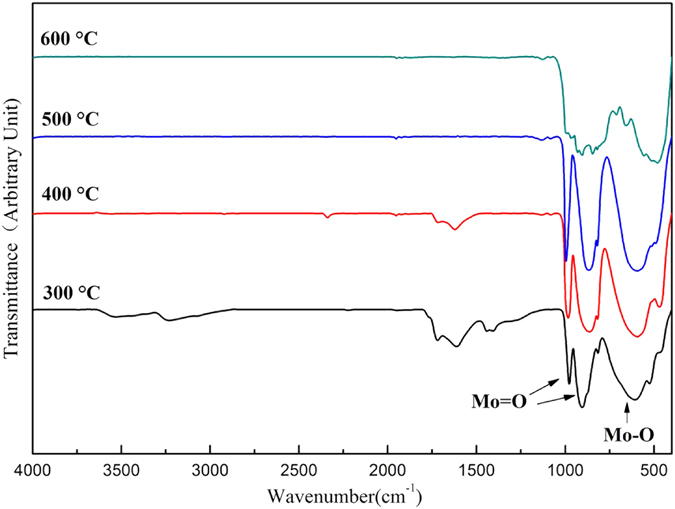
FT-IR spectrum of α-MoO_3_ synthesized at different temperature.

Figure 5 shows the surface functional groups of α-MoO_3_ products determined with FT-IR. The strong band at 996 cm^−1^ is associated with the Mo=O stretching vibration, which is an indicator for the layered orthorhombic MoO_3_ phase. The band at 867 cm^−1^ is associated with the Mo-O-Mo stretching. The band at 595 cm^−1^ is the result of the Mo_3_O single bond. Furthermore, the MoO_3_ synthesized at 300 and 400 °C shows small difference with 500 and 600 °C, for instance, the band at 1622 and 3528 cm^−1^ were attributed to the stretching of O-H groups of adsorbed water on surface of MoO_3_ synthesized at 300 °C. It suggests that the AHM was not completely decomposed at 300 °C, which is consistent with the XRD results.

### The texture properties of porous MoO_3_

**Figure 6:**
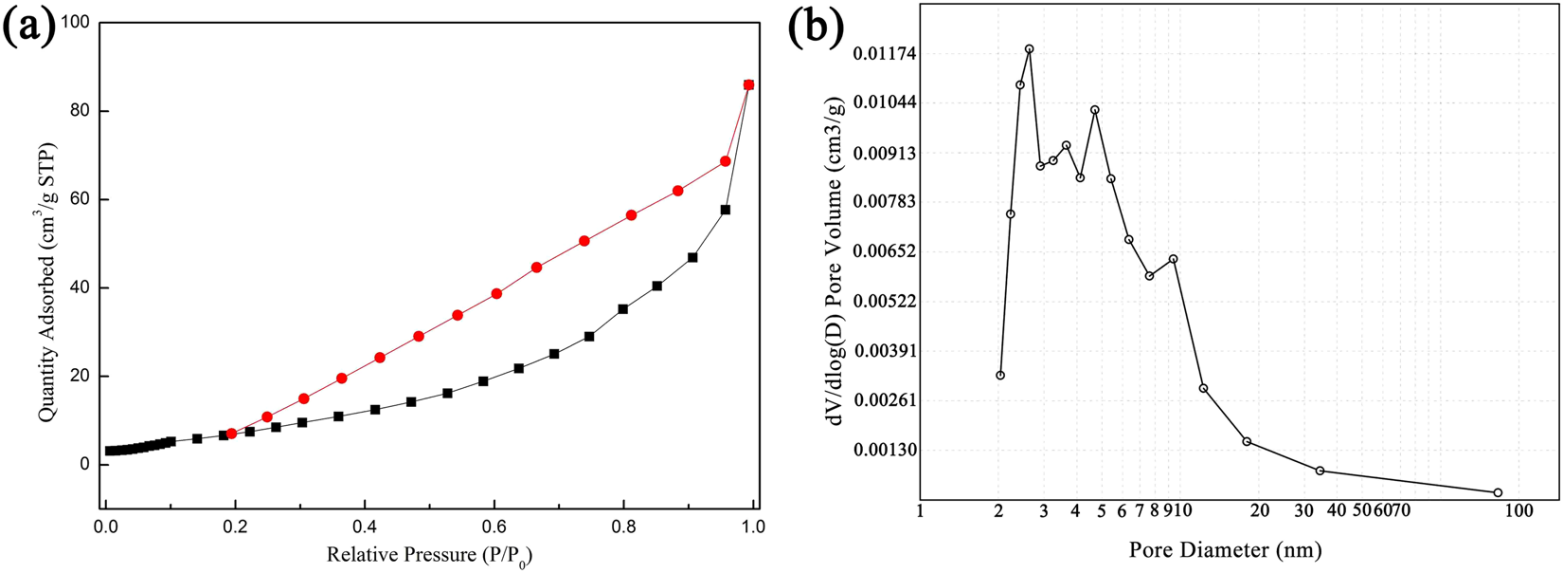
Nitrogen adsorption–desorption isotherms collected at 77 K: (**a**) Porous MoO_3_ synthesized at 400 °C, (**b**) Pore size distribution curves.

MoO_3_ porous structure was determined with N_2_ adsorption/desorption method at 77 K. The adsorption isotherms are classified as type IV-isotherms according to Brunauer-Deming-Deming-Teller (BDDT) classification (Fig. 6a). It induces that the porous structure of the monolith contains mesopores and macropores. The mesopore size distribution is illustrated in the corresponding pore size distribution in Fig. 6b. The BET surface area, pore volume and BJH desorption average pore size of monoliths synthesized at different thermal treatment temperatures are summarized in Table 2. It shows that MoO_3_ synthesized at 400 °C has the highest BET surface area of 25.62 m^2^/g and a total pore volume of 0.13 cm^3^/g. In comparison, MoO_3_ synthesized at 500 and 600 °C has low BET surface area and pore volume. It suggests that with increase of sintering temperature the MoO_3_ grains grow to micron size and results in reduction in pore volume and BET surface area.

**Table 2:** Textural properties of MoO_3_ prepared on different temperature.

**Sintering Temperature (°C)**	**BET surface area (m** ^**2**^ **/g)**	**Pore volume (cm** ^**3**^ **/g)**	**Pore size (nm)**
300	22.20	0.09	15.86
400	25.62	0.13	20.76
500	8.95	0.04	19.60
600	10.63	0.07	21.90

### XPS analysis

**Figure 7:**
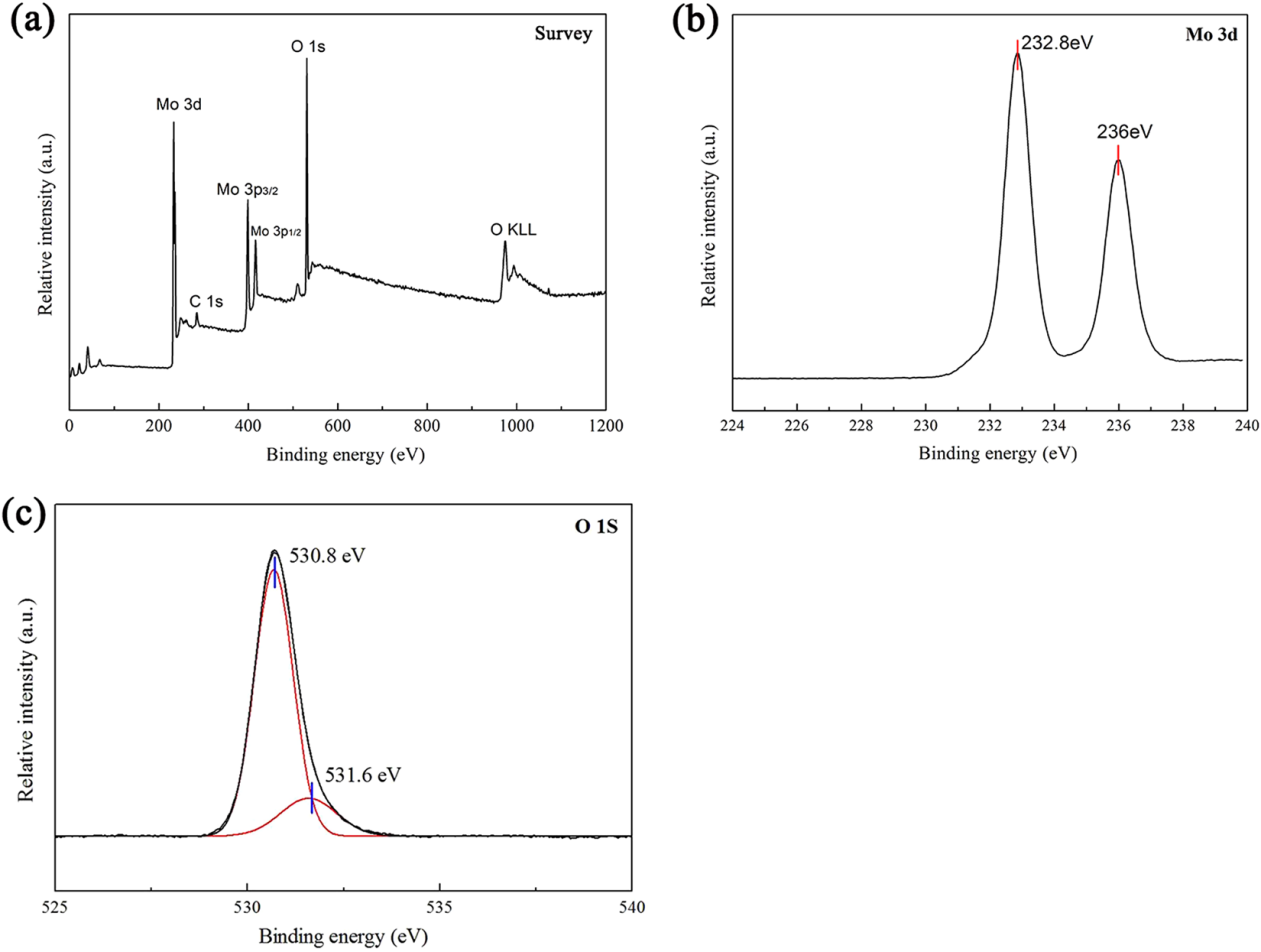
XPS spectra of the MoO_3_ synthesized at 400 °C: (**a**) A typical survey spectrum. (**b**) Mo 3d core level. (**c**) O 1 s core level.

To further study the composition and chemical state, MoO_3_ synthesized at 400 °C was analyzed by X-ray photoelectron spectroscopy (XPS) analysis. Figure 7a shows that the peaks in the spectra were assigned to Mo, O, and C. The C element results from the adventitious hydrocarbon from XPS instrument itself ^34^. No other impurities were found. The binding energies in the XPS analysis were obtained by referencing the C 1s signal at 284.5 eV. Figure 7b shows two peaks located at 232.8 eV and 236 eV can be indexed to the Mo 3d_1/2_ and Mo 3d_3/2_ signals, respectively, which can be assigned to Mo^6+^ valence state. The O 1s spectra of the sample were provided in Fig. 7c. The intense peak centered at 530.8 eV was attributed to O^2−^ anions. In addition, the binding energy at 531.6 eV belongs to hydroxyl or water molecules that are absorbed on the surface of the sample^35^.

### UV-Vis spectra analysis

**Figure 8:**
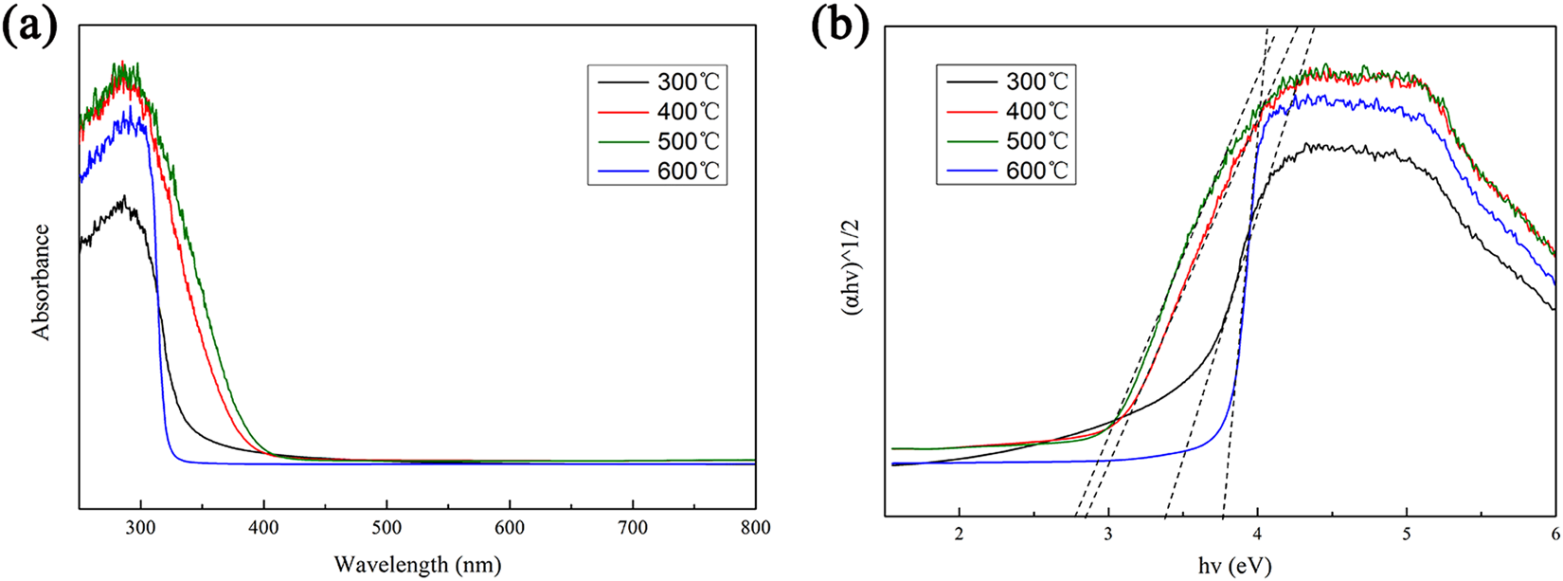
UV-Vis spectra of the as-prepared porous monoliths of MoO_3_ nanoplates.

Figure 8a shows the UV-Vis spectra collected from MoO_3_ prepared at different temperatures. When the preparation temperature was lower than 500 °C the absorbance of MoO_3_ for UV-light was increased and the absorption edge shows red shift which could be due to the grain growth and gradually increased particle size of MoO_3_. The band gaps of the samples were calculated using the Kubelka-Munk method^36^.

1 $${\rm{\alpha }}={\rm{C}}{(hv-{\rm{Eg}})}^{2}/hv$$

where C is a frequency-independent constant and α is the adsorption coefficient. The intercept from the extrapolation of the linear portion of the (α*hv*)^1/2^ ~ *hv* plot gives the band gap^37^ and is shown in Fig. 8b. The estimated band gap energy of the MoO_3_ synthesized at 300, 400, 500 and 600 °C were calculated to be 3.40, 2.85, 2.78 and 3.75 eV respectively.

### PL analysis

**Figure 9:**
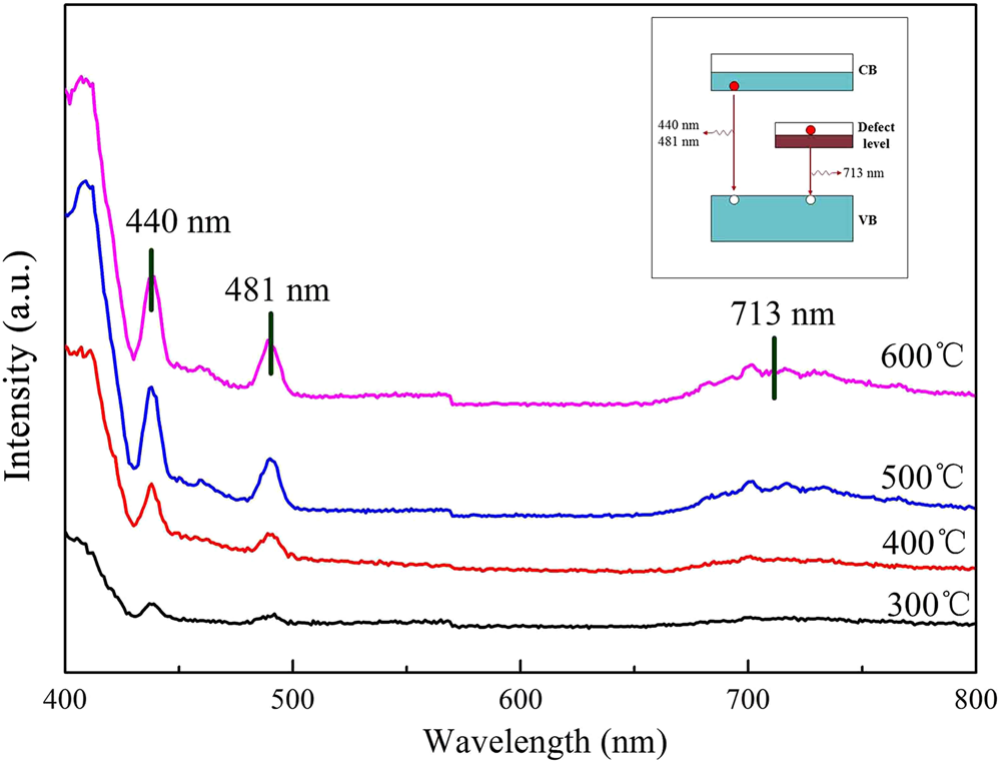
Photoluminescence spectra of MoO_3_ synthesized at different temperature.

Figure 9 shows the photoluminescence spectra of the porous MoO_3_ in the wavelength range between 400 and 800 nm under the excitation of 325 nm at room temperature. Two peaks at 440 nm and 481 nm in the emission spectrum is observed, corresponding to the recombination between the conduction bands and the valence bands. The increase of PL intensity corresponds to fast recombination of electron-hole pairs, indicating decrease of photocatalytic activity. In addition, an extra weak emission at 713 nm is observed when the temperature higher than 500 °C. The result shows the existence of a IB between the conduction bands and the valence bands. The existence of IB also led to recombination of electron-hole pairs and reduced the photocatalytic performance^38^. The relative positions among the CB, VB, and IB, as well as the two emissions are schematically shown in the inset of Fig. 9.

### Adsorption and photocatalytic properties of the samples

**Figure 10:**
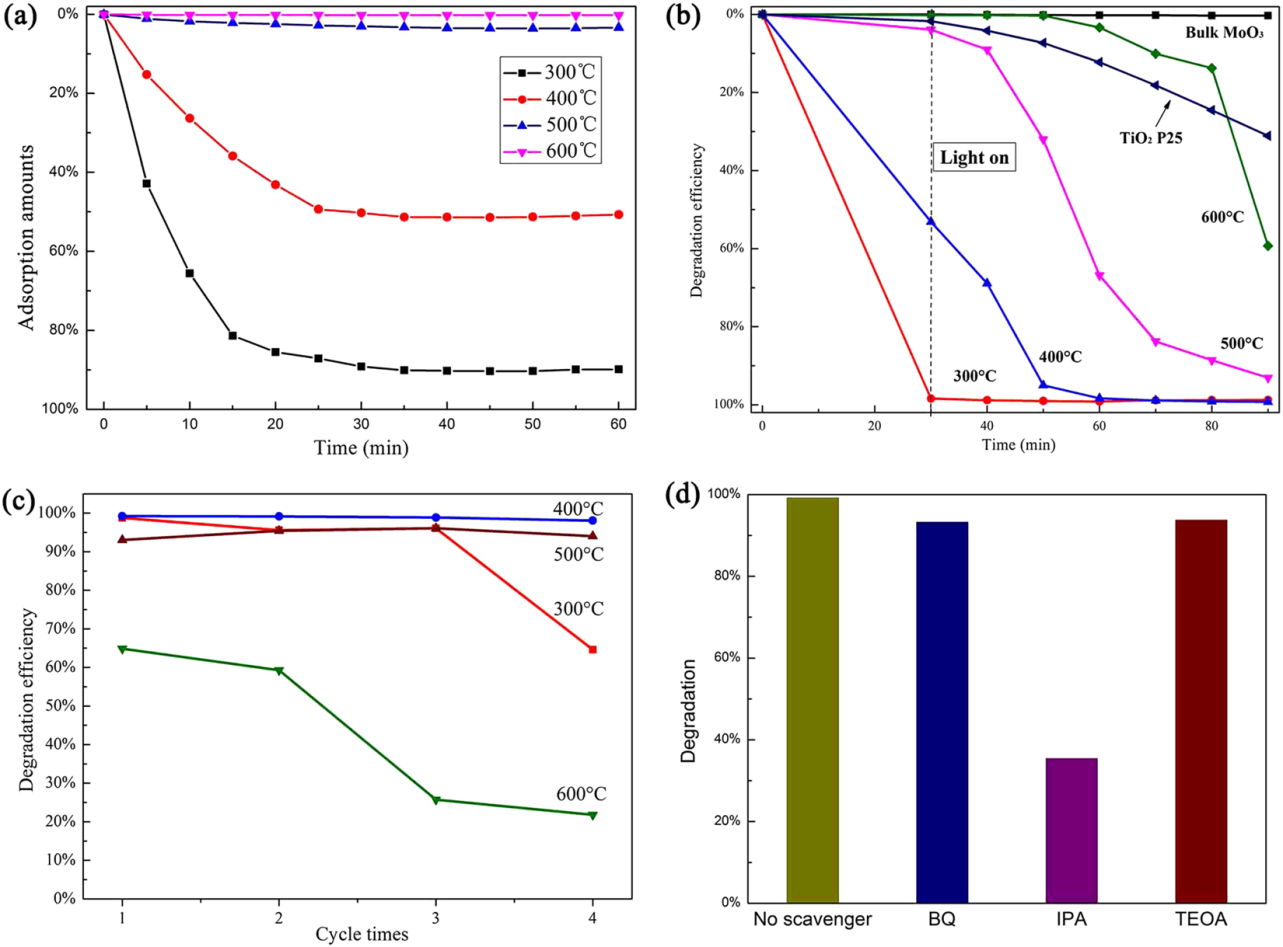
(**a**) Variation of the relative MB concentration during its adsorption over as-prepared MoO_3_. (**b**) Decolorization efficiency for the degradation of MB under visible light for first time. (**c**) Photocatalytic activity for four cycles. (**d**) Effect of different scavengers on degradation efficiency of MB.

The adsorption of contaminates molecules is a prerequisite for good photocatalytic activity^39^. Figure 10a shows the variation of the methyl blue (MB) concentration during its adsorption. It can be seen that the porous MoO_3_ established adsorption-desorption equilibrium in 30 min. In addition, the adsorption amounts of the catalysts decreased as the thermal treatment temperature increases. Figure 10b shows the photocatalytic activity of the as-synthesized MoO_3_. The as-synthesized MoO_3_ at 300 °C has the best adsorption performance and faded rate reached 98.4% after stirred for 30 min in the dark. MoO_3_ synthesized at 400 °C also has a high adsorption performance with decolorization efficiency of 53.1% under dark for 30 min and the rapid degradation efficiency with decolorization efficiency of 95.0%, 98.4% and 99.2% under visible illumination for 20, 30 and 60 min, respectively, which is higher than standard photocatalyst of TiO_2_ (P25). The stability of MoO_3_ was investigated for four cycles (Fig. 10c), and MoO_3_ synthesized at 400 °C remained active across several reaction cycles with decolorization efficiency of 98.1%, but the degradation efficiency of MoO_3_ synthesized at 300 °C decreased sharply in fourth cycle with decolorization efficiency of 64.6%. The kinetic studies of MB on MoO_3_ are calculated by using the pseudo-first-order kinetics model^40^ shown in Table 3.

2 $$\mathrm{ln}\,({C}_{{0}}/{C}_{t})=kt$$

Where *k* is the degradation rate, *C*_*0*_ is the initial concentration of MB, and *C*_*t*_ is the concentration of MB at reaction time *t* and MoO_3_ synthesized at 400 °C has the fastest reaction rate (*k* = 0.147 min^−1^). The presence of the hierarchically porous structure increases the surface area and enhances the surface adsorption of water and hydroxyl groups. Water and hydroxyl groups can react with the photo-induced holes on the surface of the catalyst to produce hydroxyl radicals, which is a strong oxidizing agent to degrade organic compounds and therefore improves the photocatalytic activity. MoO_3_ synthesized at 400 °C showed excellent photocatalytic performance and could potentially be used for photodegradation of pollutants under visible-light radiation.

**Table 3:** The degradation rate of different sintering temperature.

Sintering Temperature (°C)	300	400	500	600
*k* (min^−1^)	0.112	0.147	0.051	0.036

In order to study the mechanism of the photodegradation, the corresponding effective scavengers were added to the reaction, namely isopropyl alcohol (IPA), triethanolamine (TEOA) and benzoquinone (BQ), respectively. IPA was employed to trap ·OH, TEOA scavenges h^+^ and BQ scavenges O_2_^·−^^41^. As shown in Fig. 10d, the addition of IPA could induce the depression effect on the photodegradation of MB solution. Therefore, we can conclude that hydroxyl radicals were the main active species in the reaction systems.

### Mechanism of the photocatalytic process

**Figure 11:**
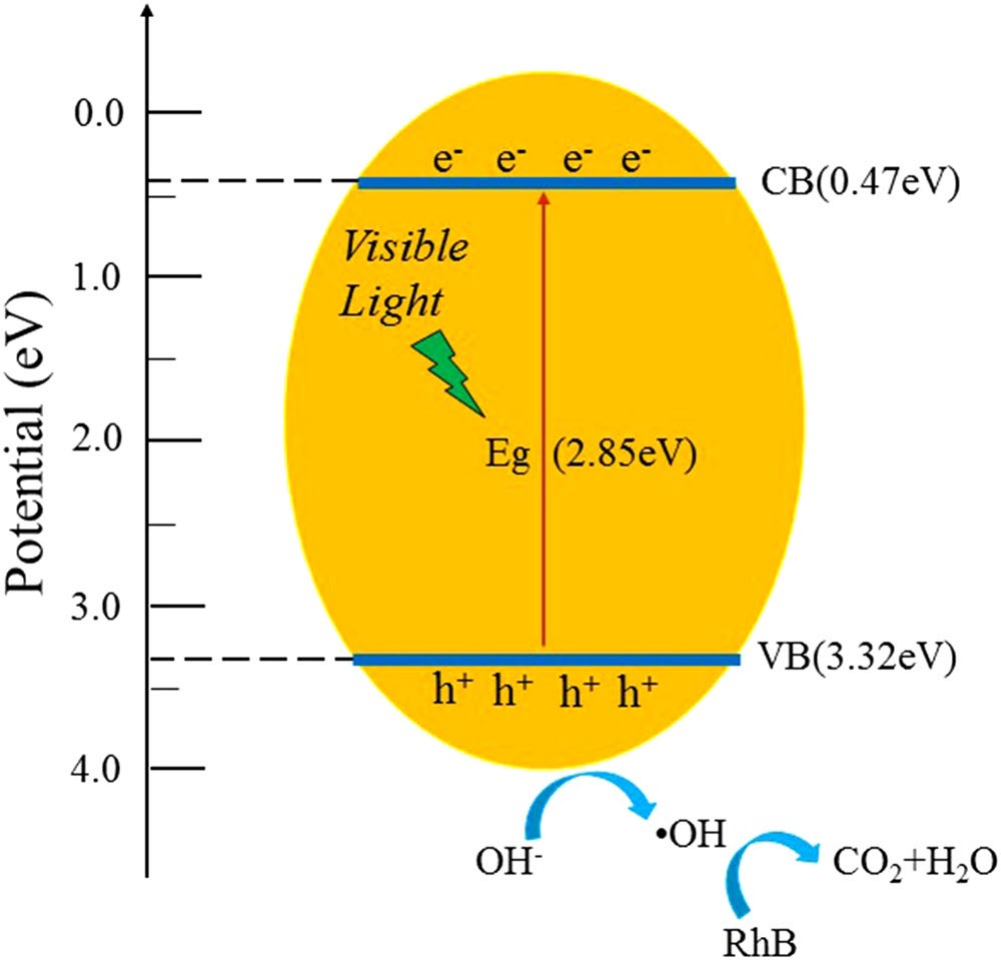
Photocatalytic mechanism of the as-prepared porous monoliths of MoO_3_ nanoplates.

According to the results of the above, we propose a possible photocatalytic mechanism of porous MoO_3_, which is illustrated in Fig. 11. The conduction band and valence band potentials of the semiconductor were calculated by the following equation^42^:

3 $${{\rm{E}}}_{{\rm{VB}}}={\rm{X}}-{{\rm{E}}}^{{\rm{e}}}+0.5{\rm{Eg}}$$

4 $${{\rm{E}}}_{{\rm{CB}}}={{\rm{E}}}_{{\rm{VB}}}-{\rm{Eg}}$$

where X is the absolute electronegativity of the semiconductor, which was defined as the geometric average of the absolute electronegativity of the constituent atoms, E^e^ is the energy of free electrons on the hydrogen scale (ca. 4.5 eV), and Eg is the band gap energy of the semiconductor^43, 44^.

Therefore, the conduction and valence band positions of MoO_3_ synthesized at 400 °C were calculated to be 0.47 eV and 3.32 eV, respectively. When MoO_3_ particles are irradiated with visible light photogenerated electrons-hole pair formed and photons can migrate to the catalyst surface and initiate redox reactions with the adsorbed H_2_O or -OH generating hydroxyl radicals (·OH)^45^. The major reaction steps in this photocatalytic mechanism are summarized as follows:

$$\begin{matrix}{{\rm{MoO}}}_{3}+hv & \to  & {{\rm{MoO}}}_{3}({{\rm{h}}}^{+}+{{\rm{e}}}^{-})\\ {{\rm{H}}}^{+}+{{\rm{H}}}_{2}{\rm{O}} & \to  & {{\rm{H}}}^{+}+\cdot {\rm{OH}}\\ {{\rm{O}}}_{2}+2{{\rm{H}}}^{+}+2{{\rm{e}}}^{-} & \to  & {{\rm{H}}}_{2}{{\rm{O}}}_{2}\\ {{\rm{H}}}_{2}{{\rm{O}}}_{2}+{{\rm{e}}}^{-} & \to  & \cdot {\rm{OH}}+{{\rm{OH}}}^{-}\\ {{\rm{h}}}^{+}+\cdot {\rm{OH}}+{\rm{MB}} & \to  & {{\rm{CO}}}_{2}+{{\rm{H}}}_{2}{\rm{O}}\end{matrix}$$

## Conclusions

High purity orthorhombic MoO_3_ was successfully synthesized by freeze-drying and subsequent thermal treatment. The thermal treatment temperature had great impact on the the morphologies and photocatalytic activity of orthorhombic MoO_3_. MoO_3_ synthesized at 400 °C had bimodal pore structure and the band gap was calculated to be 2.85 eV. The photocatalytic performance of the as-synthesized MoO_3_ monitored through photodegradation of MB under visible radiation. Compared with other temperatures, the MoO_3_ synthesized at 400 °C exhibited an excellent photodegradation activity and recyclability. The decolorization efficiency increased to 99.2% in 60 min, and the decolorization efficiency still could reach to 98.1% after four cycles.

## Experimental

### Materials

The chemical reagents were analytical grade and were used without further purification. Methylene blue (Aladdin Industrial Co., Ltd., Shanghai, China), Ammonium molybdate (AR, Tianjin Chemical Reagent Factory Co., Ltd., Tianjin, China), Polyvinyl Alcohol (86–89% hydrolyzed AR, Alfa Aesar Co., Ltd., United States).

### Preparation of porous MoO_3_

**Figure 12:**
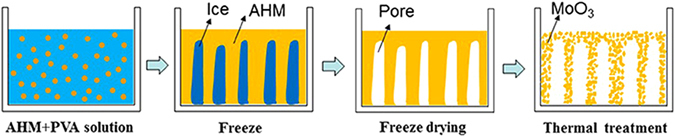
Schematic mechanisms of freeze-drying process and sintering treatment.

First, 2.5 g Polyvinyl Alcohol (PVA) was dissolved in 50.0 mL of deionized water. 1.0 g AHM was dissolved in 10.0 mL of PVA solution under heating at 80 °C in water bath. When the AHM completely dissolved, solution was poured into mould and kept for 12 h at −15 °C. Then freeze-drying was carried out at 8 Pa and −50 °C for 24 h. The freeze-dried compacts were subsequently heated in a muffle furnace at 300 °C, 400 °C, 500 °C and 600 °C for 3 h with a heating rate of 1 °C/min in air. These steps can be best seen in Fig. 12.

### Characterization of MoO_3_

The crystal structure of MoO_3_ powder was characterized by X-ray diffraction (XRD, Bruker D8 advance, Germany) with a Cu-*K*_*α*_ radiation source and settings of 30 mA and 40 KV at a scanning rate of 0.1 sec/step in the range from 5° to 70°. The surface morphology of the MoO_3_ powder was characterized by scanning election microscopy operated (SEM, Quanta 250, USA). The microstructure of the MoO_3_ powder was characterized by transmission electron microscopy (TEM, FEI Tecnai G2 F20, USA). The presence of surface functional groups on the MoO_3_ crystals were evaluated with Fourier transform infrared spectroscopy (FT-IR, Bruker vertex 80 V, Germany) analysis from 400 to 4000 cm^−1^. The Brunaner-Emmett-Teller (BET) specific surface areas of the powders were analyzed by nitrogen adsorption (JW-BK122W, China). The composition of the MoO_3_ was analysis by X-ray photoelectron spectroscopy (XPS, ESCALAB 250Xi, USA). The transmittance and reflectance spectra of the films were recorded using a UV-Vis spectrophotometer (Lambda 750s, USA) in the spectral range 300–800 nm. Photoluminescence spectra of the samples were recorded using a Fluoscence spectrophotometer (VARIAN 3000, USA).

### Photocatalytic properties characterization

Methylene blue (MB) was chosen as the representative organic pollutant to evaluate photocatalytic performance. The adsorption properties were performed in this study. 30 mg of photocatalyst powder was added to a 50 mL MB aqueous solution (30 mg/L) in the dark. During the adsorption, exactly 5 mL of suspension were taken from the reactor at given time intervals. A 150 W halogen lamp with a controlled voltage of 220 V was used as the energy source for photocatalysis. The distance between the surface of solution and the lamp was approximately 15 cm. Each 0.05 g of the as-synthesized MoO_3_ was dispersed in 100 mL of 30 mg/L MB solutions and stirred for 30 min in the dark to establish absorption-desorption equilibrium before testing. At periodic time intervals, 5 mL aliquots were sampled and ultimately centrifuged to extract particles. The percentage degradation of the dye was calculated via the following equation:

5 $${\rm{degradation}}=\frac{{C}_{{0}}-{C}_{t}}{{C}_{t}}\times 100 \% $$

Where *C*_*0*_ is initial absorbance of the dry solution before degradation, and *C*_*t*_ is absorbance of the dye solution at time *t* ^10^.
